# Temporal Course of Cerebral Autoregulation in Patients With Narcolepsy Type 1: Two Case Reports

**DOI:** 10.3389/fneur.2018.01155

**Published:** 2019-01-10

**Authors:** Zhen-Ni Guo, Xin Sun, Yingkai Zhao, Xiuli Yan, Ran Zhang, Zan Wang, Yi Yang

**Affiliations:** ^1^Clinical Trial and Research Center for Stroke, Department of Neurology, The First Hospital of Jilin University, Changchun, China; ^2^Department of Neurology, The First Hospital of Jilin University, Changchun, China; ^3^Cadre Ward, The First Hospital of Jilin University, Changchun, China

**Keywords:** narcolepsy, cerebral autoregulation, venlafaxine, fluoxetine, hypocretin

## Abstract

Cerebral autoregulation is the mechanism by which constant cerebral blood flow is maintained despite changes in arterial blood pressure. In the two presented cases, cerebral autoregulation was impaired in patients with narcolepsy type 1, and both venlafaxine and fluoxetine may have the potential to improve the impaired cerebral autoregulation. A relationship may exist between impaired cerebral autoregulation and neurological symptoms in patients with narcolepsy type 1.

## Introduction

Narcolepsy type 1, one of the two types of narcolepsy, is characterized by excessive daytime sleepiness and cataplexy with rapid and easy entry into rapid eye movement (REM) sleep (1). In patients with narcolepsy type 1, cerebral blood flow has an abnormal manifestation (2, 3), which may be involved in the progression of neurological symptoms in patients with narcolepsy type 1. However, the mechanisms remain unclear.

Cerebral autoregulation is an “intrinsic” ability of the cerebral vascular system to ensure a stable blood supply, which plays an important role in many neurologic diseases such as stroke (4–6), epilepsy (7), syncope (8), and cognitive impairment (9). The characteristics of cerebral autoregulation in narcolepsy type 1 are unknown. The mechanisms of narcolepsy type 1 are associated with hypocretin-1 deficiency. A deficiency in hypocretin neurotransmission leads to low concentrations of serotonin and norepinephrine (10–12), which are important factors for regulating cerebral autoregulation (13, 14). Thus, it is assumed that patients with narcolepsy type 1 have abnormal cerebral autoregulation, which may be an intermediate link between narcolepsy type 1 and neurological symptoms.

In the cerebral autoregulation examination and analysis, non-invasive continuous cerebral blood flow velocity and arterial blood pressure were recorded simultaneously using a transcranial Doppler and a servo-controlled plethysmograph (Finometer Model 1, FMS, Netherlands), respectively. Based on these data, the dynamic relationship between arterial blood pressure and cerebral blood flow velocity was analyzed by transfer function analysis (15). A phase difference within 0.06–0.12 Hz frequency range, derived from a transfer function, was used to evaluate cerebral autoregulation. The decreased phase difference between cerebral blood flow and blood pressure waveform represents impaired cerebral autoregulation.

This article reports the characteristics of cerebral autoregulation in two patients with narcolepsy type 1 and analyzes the therapeutic methods and potential mechanisms.

## case Report

### Case 1

**Figure 1:**
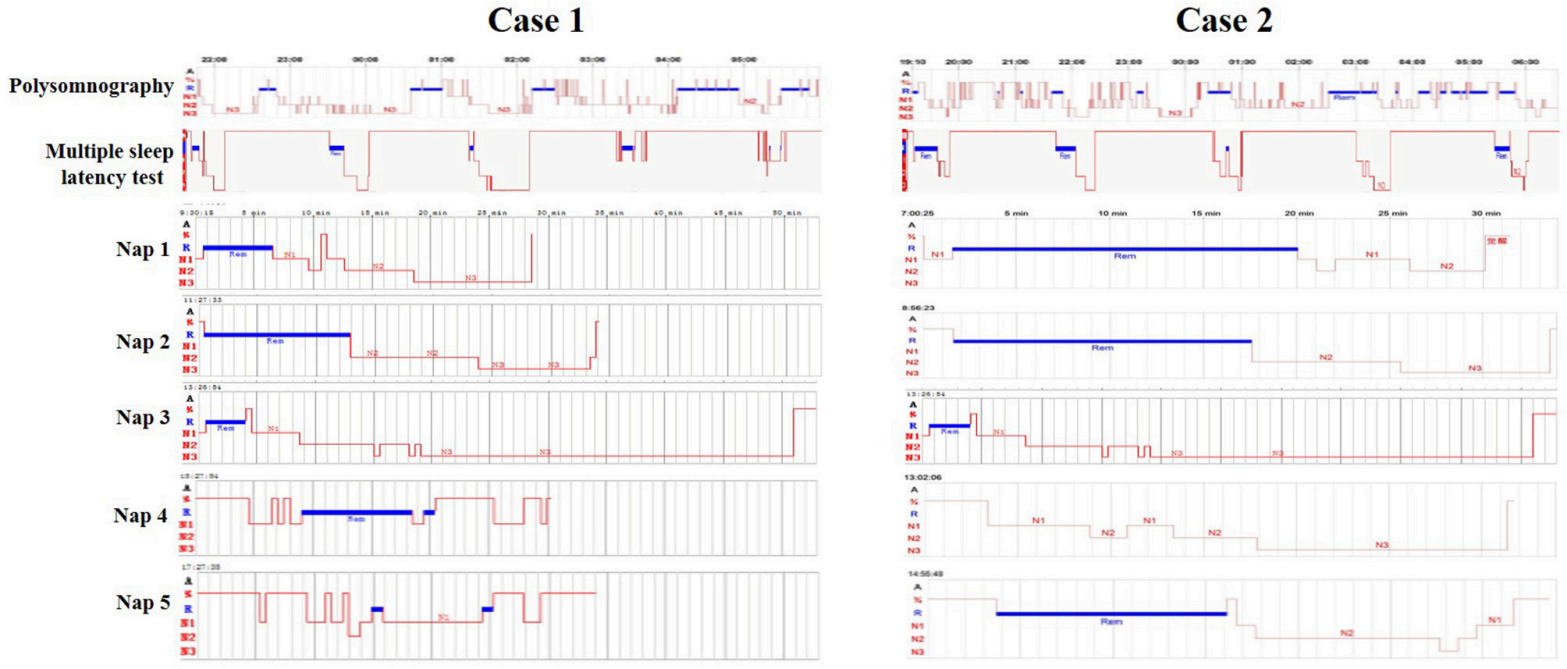
Polysomnography and multiple sleep latency tests of the two cases.

This patient was an otherwise healthy 15-years-old male. He was 1.73 m tall and weighed 65 kg. He is a junior high school student, but often could not attend class. Since the age of 12, he suffered from excessive sleepiness episodes of sudden muscular weakness triggered by laughing, visual and auditory hallucinations while falling asleep, and sleep paralysis. His Epworth Sleepiness Scale was 15. The Hamilton Rating Scale for Anxiety (HAMA) score was 5, and the Hamilton Depression Rating Scale (HAMD) was 4. Physical examination, regular laboratory examination, and brain magnetic resonance imaging findings were normal. His parents did not seek medical treatment for him before coming to our hospital. A polysomnographic examination showed that his sleep efficiency was 95.5%. Non-rapid eye movement 1 (NREM1) was 11.5%, NREM2 was 34.9%, NREM3 was 27.8%, REM was 25.9%, apnea hypopnea index (AHI) was 1.3/h, periodic limb movement disorder index (PLMDI) was 2.1/h, and REM sleep without atonia (RSWA) was observed. His multiple sleep latency test (MSLT) showed a mean MSLT sleep latency of 3.5 min, <8 min, and the presence of 5 REM sleep-onset periods while napping (Figure 1).

**Figure 2:**
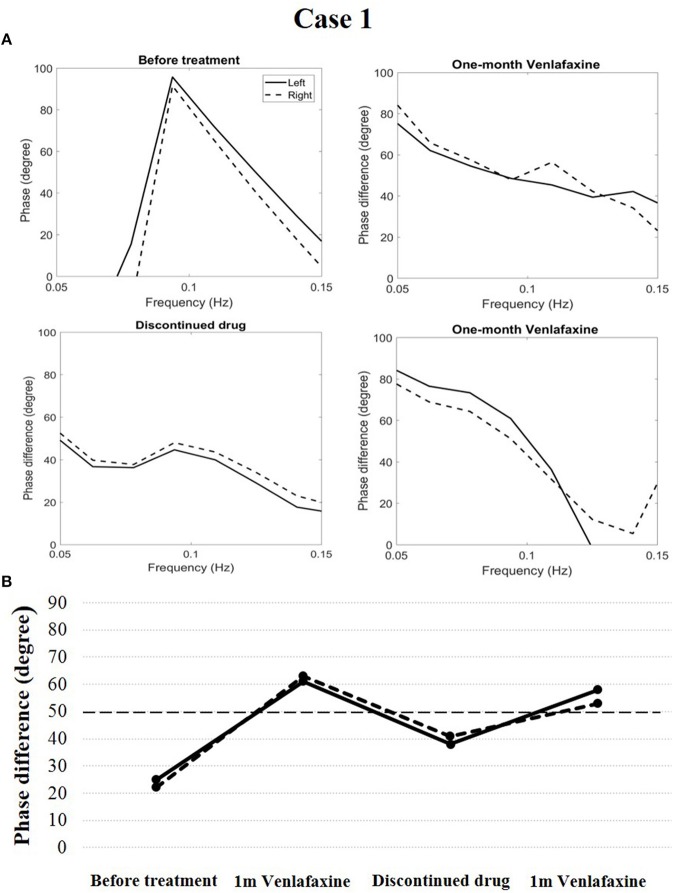
Temporal course of cerebral autoregulation in case 1. **(A)** The autoregulatory parameter (phase difference) derived from the transfer function in case 1. **(B)** The line chart shows that after 1 month of venlafaxine administration, cerebral autoregulation improved to normal. However, after the patient discontinued the drug, cerebral autoregulation deteriorated. Cerebral autoregulation improved to normal again after 1 month of venlafaxine administration.

In case 1, cerebral autoregulation before treatment showed obvious impairment. The phase difference (evaluation index of cerebral autoregulation) was 25 degrees in the left and 22 degrees in the right (reference value: 50–90 degrees in both cerebral hemispheres). The patient was diagnosed with narcolepsy type 1 and venlafaxine was administered (75 mg/d once a day in the morning). One month after treatment, his clinical symptoms were relieved, and his Epworth sleep scale was 10. His cerebral autoregulation improved and became normal (phase difference, 61 degrees in the left and 63 degrees in the right). After 6 months, the patient discontinued the drug, and his cataplexy symptoms reappeared. His Epworth sleep scale was 12. Simultaneously, cerebral autoregulation deteriorated (phase difference, 38 degrees in the left and 41 degrees in the right). The patient received venlafaxine again (75 mg/d once a day in the morning). After 1 month, his clinical symptoms were relieved, his Epworth sleep scale was 9, and his cerebral autoregulation again improved and became normal (phase difference, 58 degrees in the left and 53 degrees in the right, Figure 2).

### Case 2

This patient was an otherwise healthy 17-years-old male. He was 1.70 m tall and weighed 59 kg. He was in high school. For 1 year, he had suffered from excessive sleepiness characterized by multiple irresistible naps even when ambulating, episodes of sudden muscular weakness triggered by laughing, and visual hallucinations while falling asleep. The patient and his parents reportedly did not seek medical attention. His Epworth Sleepiness Scale was 15. His HAMA was 6 and HAMD was 4. Physical examination, regular laboratory examination, and brain magnetic resonance imaging results were normal. A polysomnographic examination showed that his sleep efficiency was 82.3%. NREM1 was 17%, NREM2 was 43%, NREM3 was 9.5%, REM was 24.5%, AHI was 1.5/h, PLMDI was 12.1/h, and RSWA was not observed. His MSLT showed a mean MSLT sleep latency of 2.6 min, fewer than 8 min, and the presence of 4 REM sleep-onset periods while napping (Figure 1).

**Figure 3:**
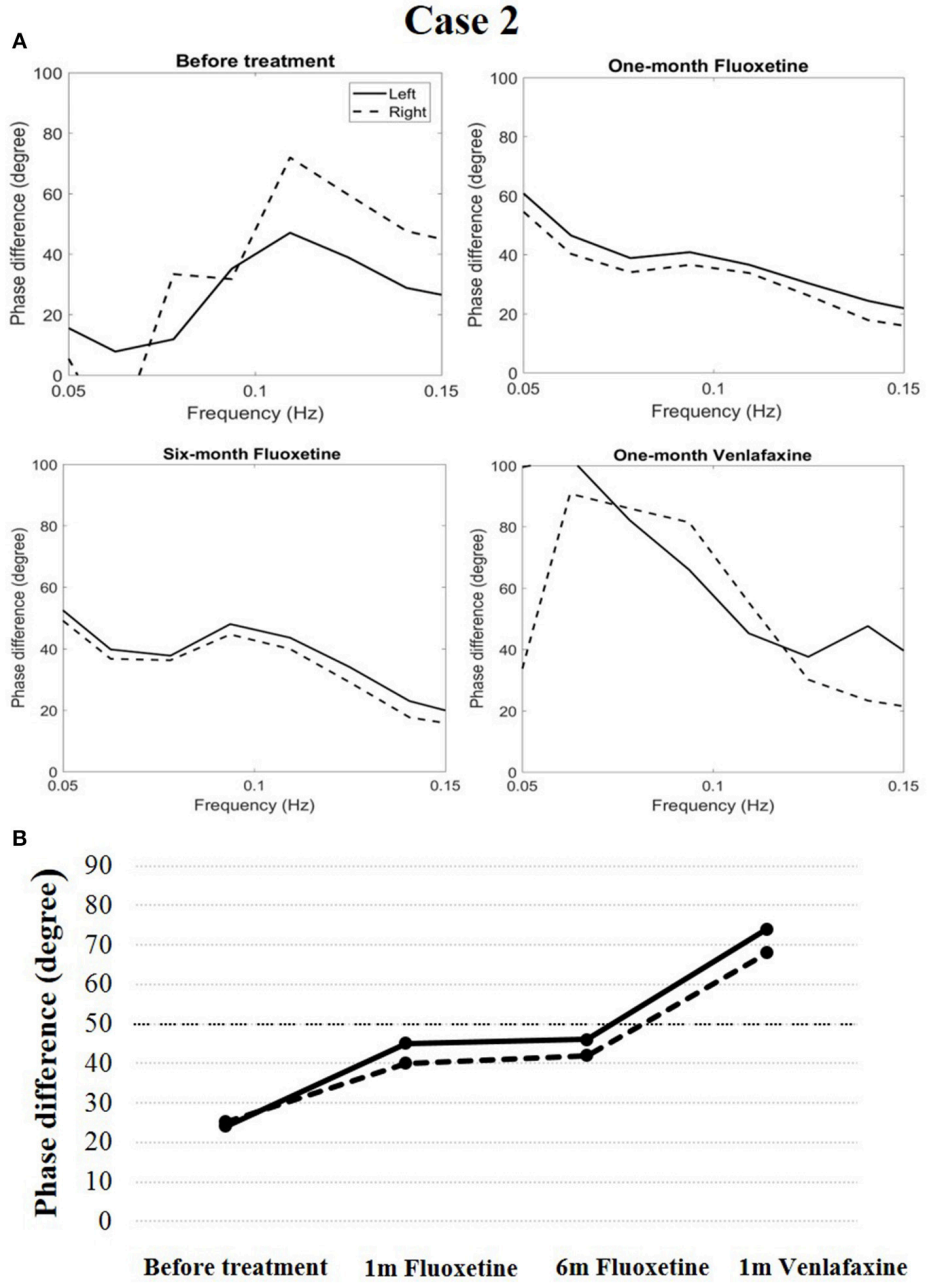
Temporal course of cerebral autoregulation in case 2. **(A)** The autoregulatory parameter (phase difference) derived from the transfer function in case 2. **(B)** The line chart shows that after fluoxetine administration, cerebral autoregulation had a tendency to rise. After 1 month of venlafaxine administration, cerebral autoregulation improved more definitively.

Cerebral autoregulation before treatment markedly decreased compared to the normal level (phase difference, 24 degrees in the left and 25 degrees in the right). The patient was diagnosed with narcolepsy type 1 and administered fluoxetine treatment (20 mg/d once a day in the morning). After 1 month of treatment, his clinical symptoms were relieved, and his Epworth sleep scale was 9. His cerebral autoregulation had the tendency to rise (phase difference, 45 degrees in the left and 40 degrees in the right). At the end of a 6-months follow-up period, his cataplexy symptoms occurred occasionally, and his Epworth sleep scale was 11. Simultaneously, the phase difference was 46 degrees in the left and 42 degrees in the right. The patient received venlafaxine (75 mg/d once a day in the morning) during the following month. His clinical symptoms were relieved, and his Epworth sleep scale was 10. His cerebral autoregulation improved clearly (74 degrees in the left and 68 degrees in the right, Figure 3).

## Discussion

Cerebral autoregulation, which is defined as the mechanism by which constant cerebral blood flow is maintained despite changes in arterial blood pressure, is critical in regulating cerebral hemodynamics and plays an important role in many neurologic diseases (3–8). In the present cases, cerebral autoregulation was obviously impaired in both patients with narcolepsy type 1, and both venlafaxine and fluoxetine improved the impaired cerebral autoregulation. Some relationships may exist between impaired cerebral autoregulation and neurological symptoms in patients with narcolepsy type 1 (such as sleep paralysis and muscular weakness), although no studies have focused on this area.

Further studies are required to understand why patients with narcolepsy type 1 present with impaired cerebral autoregulation. The hypocretin system is the major excitatory neuromodulatory system that controls the activities of the monoaminergic and cholinergic systems to control vigilance states (10, 11). In patients with narcolepsy type 1, hypocretin deficiency causes decreased function of the monoaminergic system, which leads to low levels of serotonin and norepinephrine. Previous studies have proven that norepinephrine has the function to modulate cerebral autoregulation (13). Serotonin, as a vasoactive substance, also has a potential function in regulating cerebral autoregulation (16, 17). Hence, impaired cerebral autoregulation can be detected in patients with narcolepsy type 1.

Venlafaxine belongs to the serotonin-norepinephrine reuptake inhibitor class. It can increase concentrations of serotonin and norepinephrine in both the body and brain. In case 1, after the use of venlafaxine for 1 month, the patient's cerebral autoregulation returned to normal. This may be due to the restoration of serotonin and norepinephrine concentrations. Of note, after venlafaxine treatment was interrupted, cerebral autoregulation deteriorated again. Fluoxetine is in the selective serotonin reuptake inhibitor class, and its function is to increase serotonin concentration. Theoretically, it can also improve cerebral autoregulation. In case 2, after the patient took fluoxetine for 1 month, his cerebral autoregulation had a tendency to rise, but the improvement was less than that obtained after venlafaxine administration. However, after the patient changed his treatment from fluoxetine to venlafaxine, his cerebral autoregulation improved significantly. This phenomenon indicated that the effect of venlafaxine on cerebral autoregulation may be better than that of fluoxetine.

This case report has some limitations. First, the MSLT was administered only at the first diagnosis, and there was no re-examination during the follow-up period. Second, we did not collect blood samples or cerebrospinal fluid samples to test for hypocretin (and its downstream neurotransmitters).

## Conclusions

These two cases indicated that cerebral autoregulation impairment is definitely a concomitant phenomenon of narcolepsy type 1. Both venlafaxine and fluoxetine may have the potential to improve cerebral autoregulation in patients with narcolepsy type 1.

## Ethics Statement

Written informed consent was obtained from the parents of the two patients for the publication of this case report. The protocol was approved by the ethics committee of the First Hospital of Jilin University.

## Author Contributions

Z-NG, XS, and YZ wrote the manuscript. XY and RZ prepared the figures. ZW and YY reviewed and edited the manuscript.

### Conflict of Interest Statement

The authors declare that the research was conducted in the absence of any commercial or financial relationships that could be construed as a potential conflict of interest.
